# Derivation and Validation of a Clinical and Endothelial Biomarker Risk Model to Predict Persistent Pediatric Sepsis-Associated Acute Respiratory Dysfunction

**DOI:** 10.1016/j.chstcc.2024.100120

**Published:** 2024-12-11

**Authors:** James G. Williams, Jane E. Whitney, Scott L. Weiss, Brian M. Varisco, Nadir Yehya, Mihir R. Atreya, for the Sepsis Genomics Collaborative and the Children’s Hospital of Philadelphia Sepsis Investigators

**Affiliations:** Division of Critical Care Medicine and Department of Pediatrics, University of Arkansas for Medical Sciences, Little Rock, AR; Division of Medical Critical Care, Department of Pediatrics, Boston Children’s Hospital, Harvard Medical School, Boston, MA; Division of Critical Care Medicine, Nemour’s Children’s Hospital, Wilmington, DE; Division of Critical Care Medicine and Department of Pediatrics, University of Arkansas for Medical Sciences, Little Rock, AR; Division of Critical Care Medicine, Children’s Hospital of Philadelphia, Philadelphia, PA; Division of Critical Care Medicine, Cincinnati Children’s Hospital Medical Center and Department of Pediatrics University of Cincinnati, College of Medicine, Cincinnati, OH.

**Keywords:** biomarkers, endothelial dysfunction, precision medicine, sepsis-associated respiratory dysfunction, septic shock

## Abstract

**BACKGROUND:**

Sepsis-associated ARDS results in high morbidity and mortality in children. However, heterogeneity among patients makes identifying those at risk of persistent acute respiratory dysfunction challenging. Endothelial dysfunction is a key feature of ARDS pathophysiologic characteristics, contributing to lung injury in sepsis. Incorporating endothelial biomarkers into risk models may enhance prediction of those with persistent acute respiratory dysfunction.

**RESEARCH QUESTION:**

Can clinical variables and endothelial biomarkers measured early in the course of sepsis predict risk of persistent acute respiratory dysfunction among critically ill children?

**STUDY DESIGN AND METHODS:**

This was a multicenter derivation and single center test cohort study of prospectively enrolled children with sepsis. The derivation cohort was split into training and holdout validation sets. We trained TreeNet (Minitab, LLC) and classification and regression tree (CART) models using clinical and endothelial biomarkers measured on day 1 of septic shock to predict risk of sepsis-associated acute respiratory dysfunction (SA ARD) on day 3. The performance of the CART model was tested in the holdout validation data set and in the independent test cohort.

**RESULTS:**

In the derivation (n = 625) and test (n = 162) cohorts, children with day 3 SA ARD showed increased mortality, length of mechanical ventilation, and PICU length of stay compared with those without. The TreeNet and CART models yielded comparable results. The variables included in the final CART model were presence of SA ARD on day 1, Pao_2_ to Fio_2_ ratio of < 250, soluble thrombomodulin, and vascular cell adhesion molecule 1 concentrations. This model showed an area under the receiver operating characteristic curve (AUC) of 0.88 in the training data set, sensitivity of 0.91 (95% CI, 0.86–0.94), specificity of 0.76 (95% CI, 0.68–0.82), and demonstrated reproducibility in validation data set and test cohort (AUC range, 0.78–0.83).

**INTERPRETATION:**

We derived and validated predictive models incorporating clinical and endothelial biomarkers to identify pediatric patients with septic shock at high risk of persistent acute respiratory dysfunction. Pending prospective validation, such models may facilitate enrichment and targeted intervention in future clinical trials.

Sepsis is a common cause of ARDS,^1^ with severe cases resulting in the death of every 1 in 3 affected children.^2^ Moreover, survivors experience significant long-term morbidity, including tracheostomy dependence,^3^ frequent rehospitalization,^4,5^ and a reduced quality of life.^6^ Despite this burden of disease, treatment options remain limited to antibiotics, lung protective ventilation, and organ support.^7^ Yet, clinical and biological heterogeneity among patients adds considerable complexity, underscoring the need to identify patients at high risk of persistent sepsis-associated acute respiratory dysfunction (SA ARD) who may benefit from targeted intervention(s). Yet, attempts to develop reliable risk-stratification tools for pediatric patients have been limited by sample size.^8,9^

To address this challenge, research has focused on clinical variables and candidate biomarkers, including those reflective of immune, epithelial, and endothelial pathways, to identify patients who are critically ill and at high risk of poor outcomes.^10^ Among these pathways, endothelial dysfunction plays a particularly central role in the pathophysiologic characteristics of sepsis-related organ failure. The endothelium releases cytokines and chemokines, upregulates adhesion molecules that promote leukocyte migration and inflammation, increases permeability leading to tissue hypoperfusion, and disrupts anticoagulant mechanisms, resulting in microthrombi and impaired oxygen delivery during sepsis.^1,9,11^ Furthermore, genomic studies among adults with SA ARD have implicated genes with key roles in vascular signaling^12^ and have identified candidate targets with potentially causal roles in the evolution of disease pathobiology.^13^

Given this disproportionate contribution of endothelial dysfunction to SA ARD biology, we sought to derive and validate risk-prediction models incorporating clinical variables and endothelial biomarkers early in the course of illness to reliably identify pediatric patients with sepsis at highest risk of persistent SA ARD. We leveraged a large multicenter derivation cohort of critically ill children with septic shock to train models rigorously using supervised machine learning approaches. We subsequently validated the performance of our risk model in a holdout validation data set and an independent test cohort to demonstrate prognostic usefulness and model reproducibility.

## Study Design and Methods

### Derivation and Validation Cohorts

The Sepsis Genomics Collaborative is an ongoing prospective, multicenter observational cohort of critically ill children and has been described extensively.^14–17^ The institutional review boards of 13 participating hospitals approved the study protocol (Identifier: 2008-0558; initial approval May 9, 2002). Informed consent was obtained from parent or guardian of patients. Patients in the independent test cohort were enrolled at the Children’s Hospital of Philadelphia (institutional review board identifiers: 13-010742 [approved January 19, 2014] and 13-010578 [approved July 1, 2014]). All research involving human participants was conducted in accordance with the ethical standards of the institutional review boards and with the tenets of the 1964 Declaration of Helsinki and its later amendments. Briefly, patients 0 to 18 years of age meeting consensus criteria for pediatric septic shock^18^ were recruited from PICUs across the United States between 2003 and 2023. No study-related interventions were undertaken except for blood draws and data collection. Clinical and laboratory data generated during routine care were collected from days 1 through 7, with patient outcomes tracked for 28 days. Notably, this cohort lacked chest radiography data for quantifying lung infiltrates, as well as information on noninvasive respiratory support methods and ventilator data, including mean airway pressure needed to calculate the oxygenation index (OI). Patients with missing clinical and endothelial biomarker data collected within 24 hours of meeting the study criteria were excluded from the analysis, as shown in the study flow diagram shown in e-Figure 1.

### Test Cohort

Patients were recruited prospectively through a single-center study of patients with sepsis in the PICU, some of whom met SA ARD criteria.^19^ The study was approved by the institution’s institutional review board (Identifier: 13-010578) and the study period was from 2014 through 2018. Inclusion criteria included age younger than 18 years, admission to the PICU, sepsis defined according to historical consensus criteria,^18^ with or without ARDS as defined by the Berlin criteria. Exclusion criteria included weight < 7.5 kg, WBC count < 0.5 × 10^3^/μL, known mitochondrial disease, baseline hypoxemia, cyanotic heart disease, congestive heart failure, or prolonged mechanical ventilation before enrollment. Patients were enrolled after obtaining informed consent. In this cohort, ARDS definitions were available based on Berlin criteria and details on OI. Blood was collected for plasma biomarker measurement within 24 hours of meeting study criteria and again at a second time point on days 3 through 5. Persistent SA ARD was defined as meeting the SA ARD criteria at the second time point.

### Outcomes

The primary outcome of interest was persistent SA ARD on day 3. We defined this construct as follows. Patients without any preexisting lung disease, detailed in the e-Appendix 1, were classified as having SA ARD if they met 1 of the following criteria: need for endotracheal intubation for acute respiratory failure, mechanical ventilation for > 24 hours, Pao_2_ to Fio_2_ ratio of < 250, PaCO_2_ of ≥ 65 mm Hg, or Pao_2_ of < 40 mm Hg. Patients with a preexisting lung disease were required to meet ≥ 2 of these criteria to be designated as having SA ARD.^16^ The day 3 time point was chosen because we hypothesized that prediction of patients with persistent SA ARD could allow meaningfully for timely interventions in future trials. Secondary outcomes included 28-day mortality; complicated course, that is, a composite outcome of death or ≥ 2 organ dysfunctions by day 7; and PICU length of stay (LOS) and PICU-free days, calculated by subtracting the PICU LOS from the maximum period of 28 days.

### Endothelial Biomarkers

Endothelial biomarkers measured included angiopoietin 1, angiopoietin 2, tyrosine kinase with immunoglobulin-like loops and epidermal growth factor homology domains 2, soluble thrombomodulin (sTM), IL-8, intercellular adhesion molecule 1, vascular cell adhesion molecule 1 (VCAM-1), and platelet endothelial cell adhesion molecule 1. Serum samples collected on day 1 were analyzed using Luminex assays (R&D Systems) in the derivation cohort. Plasma samples collected on day 1 were measured using a combination of Luminex and enzyme-linked immunosorbent assays in the test cohort.

### Statistical Analyses

Minitab (Minitab, LLC) version 21.1.0 software and GraphPad Prism (Graphpad Software, Inc.)version 9 software were used for data analysis and figure generation. Demographic data were summarized using numbers and percentages or medians with interquartile ranges. Differences between groups were assessed using the χ^2^ test for categorical variables and the nonparametric Kruskal-Wallis test for continuous variables. Univariable associations between predictor variables and outcomes were tested using binary logistic regression. Multivariable regression models then were constructed with 6 clinical variables: (1) patient age, (2) Pediatric Risk of Mortality III score,^18,20^ (3) presence of comorbidity, (4) immunocompromised status, (5) Pao_2_ to Fio_2_ ratio of < 250, and (6) presence of SA ARD on day 1. For this study, preexisting comorbid medical conditions were categorized by the organ system affected (eg, heart, lung, neurologic, kidney, gastrointestinal or liver, endocrine, hematologic, cancer, bone marrow, and solid organ transplantation). This was coded as a binary variable indicating the presence (yes) or absence (no) of comorbidity. Candidate endothelial biomarkers were selected based on independent association with the primary outcome using backward elimination with an α value of .1. A 2-tailed *P* value of < .05 was considered statistically significant.

### Risk Prediction Modeling

Patients in the derivation cohort were split randomly in a 60:40 ratio into training and holdout validation data sets. We chose this approach, rather than splitting based on year of enrollment or study site, to ensure that the models were exposed to a representative mix of data, improving their ability to generalize to new and unseen data. We used TreeNet (Minitab), a stochastic gradient boosting method, and classification and regression tree (CART) to develop predictive models for day 3 SA ARD (using clinical variables and select endothelial biomarkers. TreeNet builds multiple CART trees iteratively, using residuals from previous trees to enhance predictive accuracy and to capture subtle patterns. However, TreeNet models are less interpretable because contributions are distributed across many iterations and the black box nature of the algorithms used. In contrast, CART provides clear decision rules, revealing how specific combinations of predictors influence outcomes. Thus, both methods are complementary: TreeNet enhances accuracy and generalization by capturing complex patterns, whereas CART aids in identifying key interactions and provides actionable decision rules.

For TreeNet models, we did not apply class weights and allowed interactions of all orders. A total of 300 trees were grown, with a minimum of 3 and a maximum of 6 cases per terminal node. The optimal number of trees was selected based on minimum misclassification rate. To mitigate overfitting, a learning rate of 0.01 was used. For CART models, we also did not use class weights. Node splitting was based on the class probability method, and we selected the optimal tree within k = 1 SEs of the minimum misclassification cost. A minimum of 15 cases per terminal node was required for CART models.

### Validation

**Figure 1 –:**
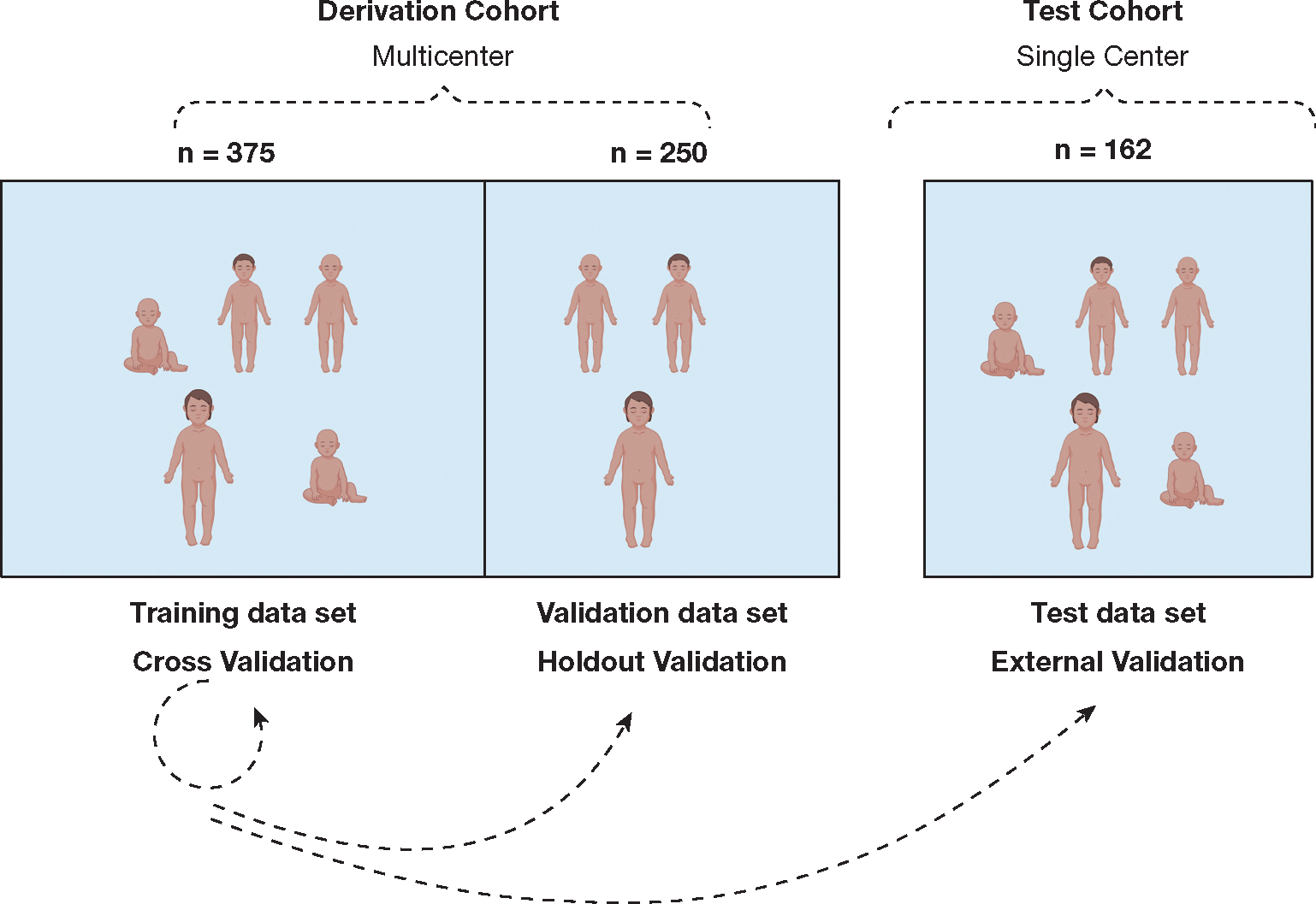
Diagram showing overview of study procedures. The derivation cohort (n = 625) comprised a prospective multicenter observational study of pediatric patients with septic shock. The test cohort (n = 162) comprised data from a prospective single-center study of pediatric patients with sepsis. The derivation cohort was split in a 60:40 ratio into training and holdout validation sets. Stochastic gradient boosting using TreeNet and classification and regression tree (CART) analyses were used in the training set. The performance of the clinical and endothelial biomarker CART model derived then was tested in the holdout validation data set and test cohorts.

Because of congruence in outputs between TreeNet and CART models in the derivation cohort, we selected the CART model for validation, prioritizing its simplicity, interpretability, and practicality for clinical application. We used a 10-fold cross-validation approach within the training data set. Only the CART model was tested in the holdout validation data set of the derivation cohort and in the independent test cohort, stratifying patients as high-risk or low-risk for day 3 SA ARD. Clinical outcomes were compared across patient risk strata to assess prognostic usefulness and reproducibility. The overall study structure and scheme for validation are illustrated in Figure 1.

### Model Performance Metrics

Model performance was evaluated using the area under the receiver operating characteristic curve (AUC), which measures the model’s ability to discriminate between high-risk and low-risk patients, as well as diagnostic test characteristics. Model accuracy and reliability was determined by Brier scores, which were calculated with the R (The R Project for Statistical Computing) package scoring (Ed Merkle). Brier scores range from 0 to 1, with lower scores indicating better calibration, which means that predicted probabilities aligned closely with observed outcomes.

## Results

### Clinical Characteristics of Patients With and Without Persistent SA ARD

The demographic features, clinical characteristics, and outcomes of interest in patients with (n = 219) and without (n = 156) day 3 SA ARD in the training data set are presented in Table 1. Patients with day 3 SA ARD were younger, less commonly were female, and had a higher illness severity at disease onset. Patients with day 3 SA ARD experienced worse clinical outcomes, including 28-day mortality, complicated course, longer PICU LOS, and fewer PICU-free days compared with those without day 3 SA ARD. Those with day 3 SA ARD also were more likely to remain intubated, to require mechanical ventilation, and to require continuous renal replacement therapy on day 7 of illness compared with those without day 3 SA ARD. Patients with day 3 SA ARD were more likely to have a pulmonary source of infection compared with those without.

### Association Between Predictor Variables and Persistent SA ARD

Univariable and multivariable associations between predictor variables and day 3 SA ARD in the training data set are detailed in e-Tables 1 and 2. Age, Pediatric Risk of Mortality III scores, presence of comorbidity, immunocompromised status, Pao_2_ to Fio_2_ ratio of < 250, and presence of SA ARD on day 1 all were associated with day 3 SA ARD. Increments in angiopoietin 2, angiopoietin 2 to angiopoietin 1 ratio, angiopoietin 2 to tyrosine kinase with immunoglobulin-like loops and epidermal growth factor homology domains 2 ratio, IL-8, sTM, platelet endothelial cell adhesion molecule 1, and intercellular adhesion molecule 1 were associated with increased odds of day 3 SA ARD, whereas increments in VCAM-1 were associated with a decreased odds of day 3 SA ARD. In multivariable models with adjustment of clinical variables in the models, sTM, VCAM-1, and angiopoietin 1 remained independently associated with outcome of interest.

### Derivation of Risk Prediction Models Including Clinical Variables and Endothelial Biomarkers for Persistent SA ARD

Model summary and confusion matrix of a TreeNet model including only clinical variables predictive of day 3 SA ARD in the training data set are detailed in e-Tables 3 and 4, with relative variable importance shown in e-Figure 2. The TreeNet model incorporating endothelial biomarkers predictive of day 3 SA ARD is detailed in e-Tables 5 and 6 and e-Figure 3. This model had an AUC of 0.96 (95% CI, 0.95–0.98) and 0.87 (95% CI, 0.83–0.90) on 10-fold cross-validation (e-Fig 4). Although the model inclusive of biomarkers showed comparable sensitivity (93.15% vs 93.61%) and specificity (73.7% vs 69.2%) as models without biomarkers, the misclassification and false-positive rates were lower with inclusion of the biomarkers. The biomarkers in a decreasing order of relative importance were sTM, VCAM-1, and angiopoietin 1, with respective 1-predictor partial dependence plots shown in e-Figure 5. Notably, we identified that patients with low sTM and high VCAM-1 concentrations had a lower odds of day 3 SA ARD in the training data set, as illustrated in the surface plot in e-Figure 6.

**Figure 2 –:**
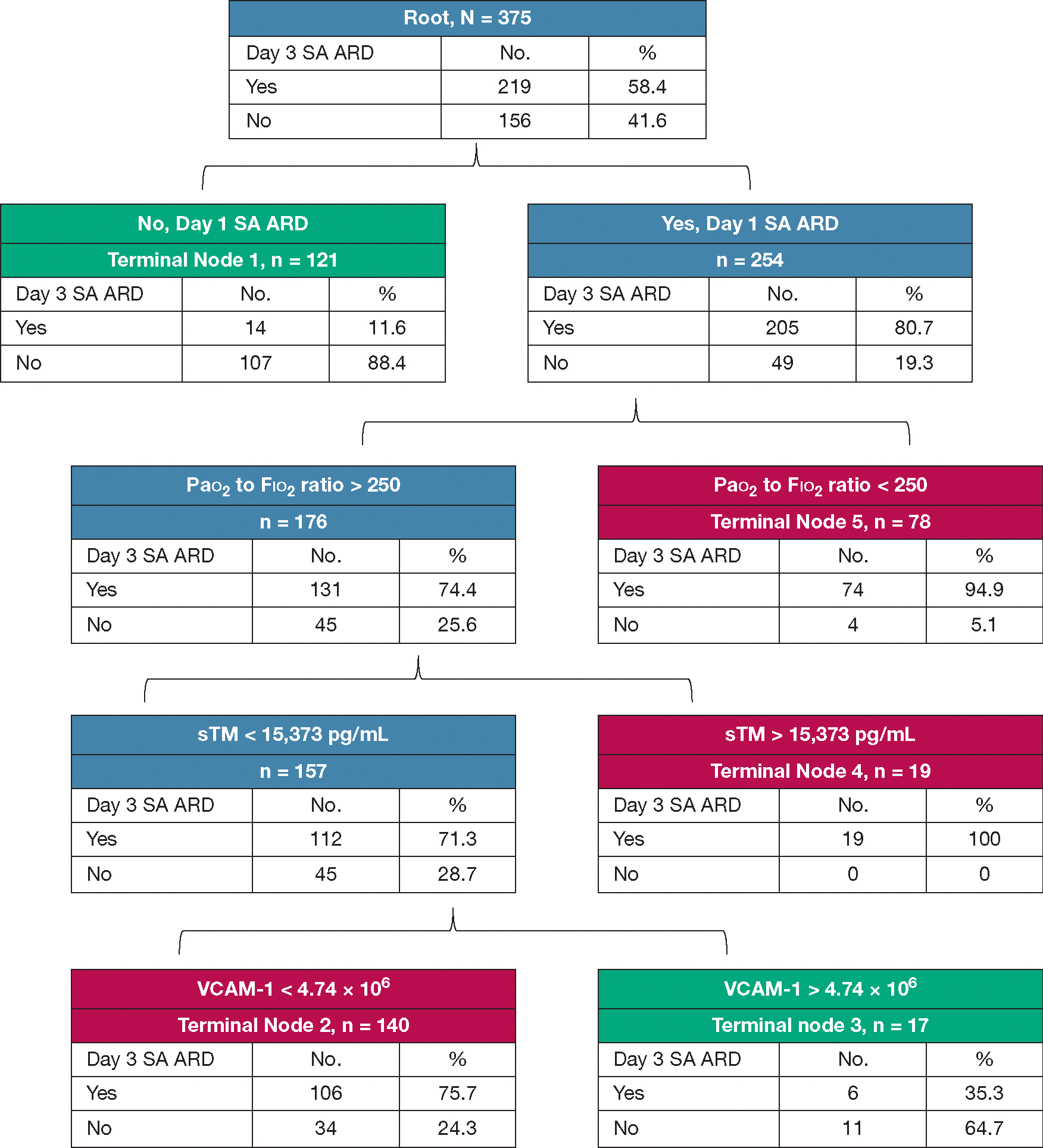
Classification and regression tree showing the clinicoendothelial biomarker model to stratify patients based on risk of day 3 sepsis-associated acute respiratory dysfunction (SA ARD). The root node at the top of the tree consisted of all patients in the training data set, of whom 219 patients had day 3 SA ARD. The top decision point was based on the presence of SA ARD on day 1. Patients who did not have day 1 SA ARD were unlikely to have day 3 SA ARD (terminal node 1, 11.6%). Patients with day 1 SA ARD and Pao_2_ to Fio_2_ ratio of < 250 had a high rate of day 3 SA ARD (terminal node 5, 94.9%). Among patients who had day 1 SA ARD but a Pao_2_ to Fio_2_ ratio of > 250, all patients with sTM concentrations of > 15,374 pg/mL had day 3 SA ARD (terminal node 4, 100%). Among patients with sTM concentrations of ≤ 15,374 pg/mL, patients with VCAM-1 concentrations of < 4.7 × 10^6^ pg/mL showed a higher rate of day 3 SA ARD (terminal node 2, 75.7%). In contrast, those with VCAM-1 concentrations of > 4.7 × 10^6^ pg/mL showed a relatively low rate of day 3 SA ARD (terminal node 3, 35.3%). Patients assigned to terminal nodes 1 and 3 were considered to belong to low-risk strata and those assigned to terminal nodes 2, 4, and 5 were considered to belong to high-risk strata. SA ARD = sepsis-associated acute respiratory dysfunction; sTM = soluble thrombomodulin; VCAM-1 = vascular cell adhesion molecule 1.

**Figure 3 –:**
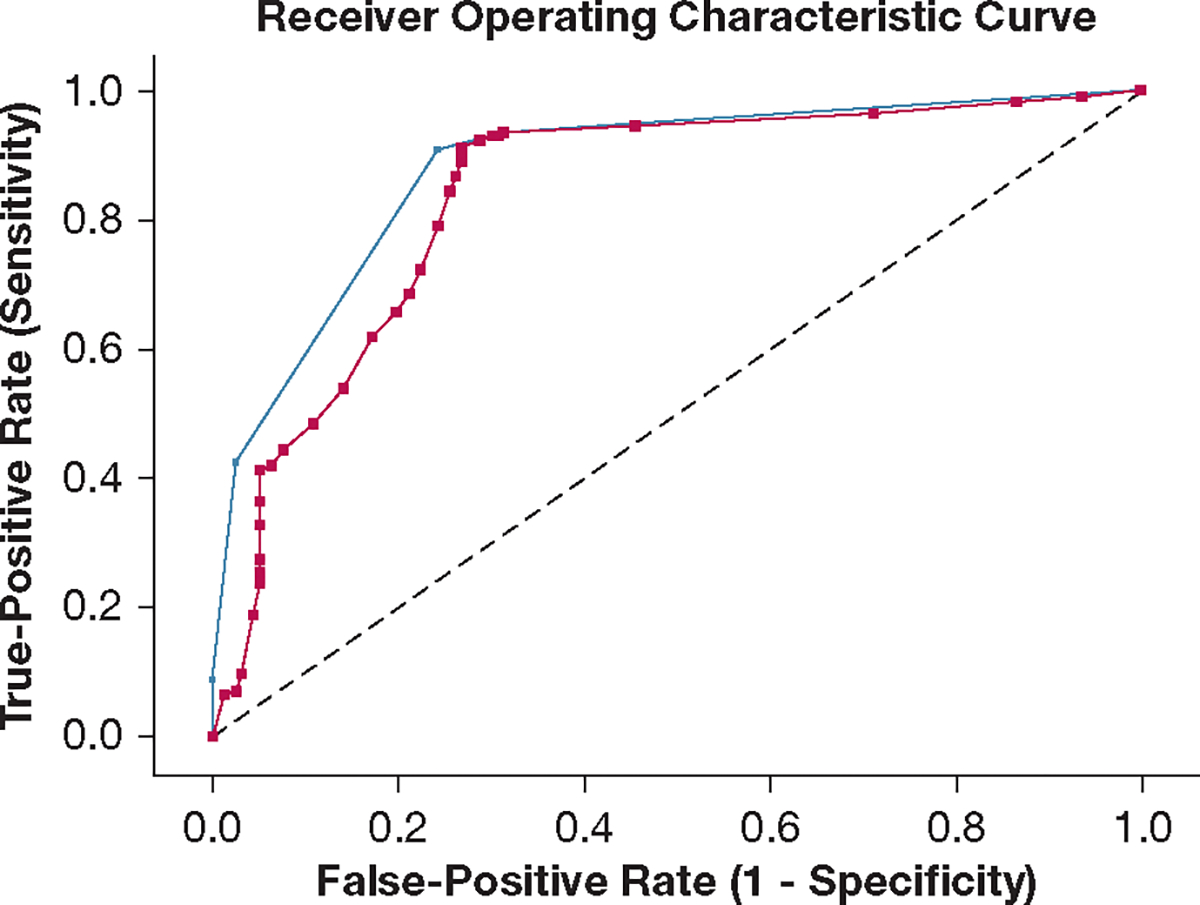
Graph showing area under the receiver operating characteristic curve for the classification and regression tree model predictive of day 3 sepsis-associated acute respiratory dysfunction in the training dataset (blue, 0.88) and on 10-fold cross-validation (black, 0.83).

Figure 2 shows the CART model predictive of day 3 SA ARD in the training data set. Consistent with the TreeNet model, the presence of day 1 SA ARD and Pao_2_ to Fio_2_ ratio of < 250 were key decision points at the top of the tree. Patients who did not have day 1 SA ARD were unlikely to have day 3 SA ARD (terminal node 1, 11.6%). Patients with day 1 SA ARD and Pao_2_ to Fio_2_ ratio of < 250 had a high rate of day 3 SA ARD (terminal node 5, 94.9%). Among patients with day 1 SA ARD but a Pao_2_ to Fio_2_ ratio of > 250, all patients with sTM concentrations of > 15,373 pg/mL had day 3 SA ARD (terminal node 4, 100%). Among patients with sTM concentrations of ≥ 15,374 pg/mL, patients with VCAM-1 concentration < 4.7 × 10^6^ pg/mL had a higher rate of day 3 SA ARD (terminal node 2, 75.7%). In contrast, those with VCAM-1 concentration of > 4.7 × 10^6^ pg/mL had a relatively low rate of day 3 SA ARD (terminal node 3, 35.3%). The AUC of the CART model inclusive of endothelial biomarkers was 0.88 and was 0.83 on 10-fold cross-validation in the training data set (Fig 3). The model showed comparable sensitivity of 90.8% (95% CI, 86.1%-94.2%) vs 93.6% (95% CI, 89.2%-96.3%) and higher specificity of 75.6% (95% CI, 68.0%-81.2%) vs 68.5% (95% CI, 60.6%-75.6%) as compared with CART models without endothelial biomarkers (data not shown). Brier scores for the TreeNet and CART models in the training data set were 0.147 and 0.154, respectively.

### Validation and Testing of Risk Prediction Models Including Clinical Variables and Endothelial Biomarkers for Persistent SA ARD

We assessed the performance of the CART model for day 3 SA ARD among 250 patients in the holdout validation set. The AUC of the model to predict day 3 SA ARD was 0.79 (e-Fig 7), with a positive predictive value of 84% and a negative predictive value of 74%. Model Brier score in this data set was 0.197. Comparisons of clinical characteristics and outcomes by risk strata in the holdout validation set are shown in e-Table 7. One hundred fifty-six patients (62.7%) were designated as high risk based on assignment to terminal nodes 2, 4, and 5, and 93 patients (37.3%) were designated as low risk for day 3 SA ARD based on assignment to terminal nodes 1 and 3. Patients deemed high risk for day 3 SA ARD were more likely to have a complicated course, longer PICU LOS, and fewer PICU-free days. One hundred thirty-one patients (85.1%) classified as high risk actually had day 3 SA ARD compared with 21 patients (30.0%) in the low-risk group (*P* < .001). No differences in mortality, rate of sepsis-associated acute kidney injury, or need for continuous renal replacement therapy were found between risk strata.

The test cohort comprised 162 pediatric patients admitted to the PICU with sepsis. The demographic and clinical characteristics of this cohort based on the presence of persistent SA ARD are presented in e-Table 8. The rate of persistent day 3 SA ARD by terminal node of the CART model in the test cohort is shown in e-Figure 8. As shown, among 90 patients classified as high risk based on the CART model (terminal nodes 2, 4, and 5), 58 patients (64.5%) had day 3 SA ARD in the test cohort relative to 10 of 68 patients (14.7%) among those classified as low risk (terminal nodes 1 and 3). Table 2 shows outcomes of patients classified as high risk and low risk of persistent SA ARD in the test cohort. Notably, those classified as being at high risk of persistent SA ARD showed a greater need for mechanical ventilation on day 3 (71.1% vs 38.9%; *P* < .001) and day 7 (43.3% vs 15.2%; *P* = .007) and included a higher proportion of patients with a Pao_2_ to Fio_2_ ratio of < 250 at the latter time point (17% vs 0%; *P* = .048). Although the OI was numerically higher among patients classified as being at high risk, no statistically significant differences were found between the groups at either day 3 or 7 in the test cohort. The AUC of the CART model in the test cohort was 0.83 (e-Fig 9). The CART model had a Brier score of 0.14 in the test cohort. The diagnostic test characteristics of the CART model inclusive of clinical variables and endothelial biomarkers in the training, validation, and test data sets are summarized in Table 3.

## Discussion

Our study demonstrated a significant association between persistent SA ARD and poor clinical outcomes in a large cohort of critically ill children with septic shock. We developed and validated predictive models using clinical data and endothelial biomarkers measured on day 1 of pediatric septic shock to identify reliably patients at risk of persistent SA ARD by day 3. The CART model, which was tested in a holdout validation data set and an independent cohort, showed prognostic usefulness and reproducibility.

Recent studies have focused on developing risk models predictive of acute hypoxemic respiratory failure among adults with sepsis.^21–23^ These models included combinations of between 6 and 13 clinical variables, laboratory values, and select biomarkers including IL-6, with AUCs ranging from 0.71 to 0.81. Studies predictive of SA ARDS and related outcomes among children has been relatively limited. Whitney et al^24^ prospectively validated a biomarker-based risk model^8^ consisting of patient age, C-C chemokine ligand 3, heat shock protein family A member 1B, and IL-8 to predict mortality-risk in pediatric ARDS. This model’s performance was evaluated at 3 different time points with an AUC ranging from 0.68 to 0.78.

Few pediatric studies have focused on establishing the association between endothelial biomarkers and risk of SA ARDS outcomes. Monteiro et al^25^ demonstrated that sTM predicted mortality risk and extrapulmonary organ failure in children with ARDS in the Randomized Evaluation of Sedation Titration for Respiratory Failure (RESTORE) trial. Members of our group have shown that angiopoietin 2 to angiopoietin 1 ratio, VCAM-1, and von Willebrand factor could discriminate between direct and indirect lung injury in pediatric sepsis.^9^ Patients with indirect or extrapulmonary sepsis meeting criteria for ARDS had elevated levels of angiopoietin 2, angiopoietin 2 to angiopoietin 1 ratio, von Willebrand factor, and endocan relative to those without ARDS and were associated with higher rates of a complicated course.^26^

In our study, the inclusion of commonly available clinical measures along with selected biomarkers helped to identify patients at risk of persistent SA ARD. The biomarkers added specificity to the model by identifying a subset of patients with less severe day 1 SA ARD (defined by a Pao_2_ to Fio_2_ ratio of > 250) who nevertheless would meet criteria for persistent SA ARD. Such subsets characterized by high sTM concentrations or relatively low sTM concentrations and low VCAM-1 concentrations may represent a potential subgroup for enrichment in clinical trials for interventions aimed at halting disease progression, as underscored by the Pediatric Acute Lung Injury Consensus Conference guidelines.^27^

Among the endothelial biomarkers tested, sTM—a cleavage product from the vascular endothelium, where it serves as a major component of the endogenous anticoagulation system^28^—showed the highest odds of day 3 SA ARD, aligning with findings from previous literature.^25,29–31^ Pending validation, our risk predictive model may help to enrich patients in future clinical trials of therapies targeting endothelial dysfunction such as recombinant human sTM.^31^ Another intriguing finding in our study is the protective association between VCAM-1 and day 3 SA ARD outcomes. This was particularly evident among patients with relatively low sTM concentrations, suggesting a complex interaction, which merits further investigation.

Our study has several limitations. First, patients were included in the derivation cohort between 2003 and 2023. However, only a total of 53 patients were recruited between 2003 and 2013. Sensitivity analyses excluding these patients did not change associations nor risk prediction models. All patients in the test cohort were recruited between 2014 and 2018. Nonetheless, validation in more contemporary data sets is necessary. Second, the lack of ventilator and chest radiograph data in patients in the derivation cohort precluded designations of ARDS based on formal criteria. We mitigated this by testing our model in a test cohort in which ARDS was defined based on Berlin definitions with comparable model performance. However, the number of patients with OIs available was limited, in turn limiting our ability to identify statistically significant differences in OI based on risk strata in the test cohort. Third, the choice of biomarkers was limited to those previously measured and readily available in the respective cohorts. Although broadly thought to serve as a surrogate for endothelial activation, several of the markers (IL-8, intercellular adhesion molecule 1, and VCAM-1) also are produced extensively by activated leukocytes. Future research that includes putative biomarkers of other compartments including the respiratory epithelium may enhance model performance and specificity.^32–35^ Fourth, the endothelial biomarkers selected for the final models were those that remained significant for the outcome of interest after adjusting for all clinical variables. It is likely that other relevant biomarkers that influence factors like illness severity and the risk of persistent SA ARD may have been excluded from the predictive models. This is because their effects likely are subsumed by the clinical variables, which inherently capture much of the risk that these biomarkers represent. Consequently, although these biomarkers likely are highly biologically important, their predictive value may have been overshadowed by stronger clinical predictors in the modeling process.

## Interpretation

We derived and validated a clinical and endothelial biomarker risk model to facilitate the early identification of patients at risk of persistent SA ARD, a condition associated with considerable mortality and morbidity in this cohort. Pending prospective validation, such an integrated approach may inform the enrichment of patients in clinical trials and targeted therapeutic interventions that seek to mitigate the risk of SA ARD.

## Supplementary Material

MMC1

MMC2

fig5

fig1

fig4

fig9

fig2

fig7

fig3

fig8

fig6

## Figures and Tables

**TABLE 1 ]:** Demographic Data and Clinical Characteristics of Patients With and Without Day 3 SA ARD in the Training Data Set

Variable	Day 3 SA RD		*P* Value

	Yes (n = 219)	No (n = 156)	

Age, y	3.1 (0.8–7.4)	6.5 (3.3–13.0)	< .001
Sex, female	95 (43.4)	86 (55.2)	.025
Race (self-identified)			.370
White	157 (71.7)	117 (75.0)	
Black	30 (13.7)	14 (9.0)	
Other^a^	32 (14.6)	25 (16.0)	
Ethnicity (self-identified)			.425
Hispanic or Latino	33 (15.1)	19 (12.2)	
PRISM III score	12 (6–19)	9 (5–14)	.003
28-d mortality	43 (19.6)	4 (2.6)	< .001
Complicated course	121 (55.3)	9 (5.8)	< .001
PICU LOS	8 (5–15)	3 (1–7)	< .001
PICU-free days	20 (13–23)	25 (21–27)	< .001
Day 7 MV	113 (71.5)	8 (17.8)	< .001
Day 7 intubated	61 (38.6)	0 (0)	< .001
Day 7 Pao_2_ to Fio_2_ ratio < 250	33 (20.9)	0 (0)	< .001
Day 7 CRRT^b^	24 (11.0)	3 (1.9)	< .001
Day 7 percent fluid overload d 1–7	6.9 (−0.5 to 15.2)	3.6 (0–8.6)	.023
Day 1–7 cardiac arrest	43 (19.6)	4 (2.6)	< .001
Positive blood culture findings	37 (16.9)	31 (19.9)	.461
Positive culture findings	137 (62.6)	80 (51.3)	.029
Source			< .001
Pulmonary	69 (31.5)	17 (10.9)	
Extrapulmonary	68 (31.1)	63 (40.4)	
Steroids	124 (56.6)	72 (46.2)	.045

Data are presented as No. (%) or median (interquartile range) unless otherwise indicated. CRRT = continuous renal replacement therapy; LOS = length of stay; MV = mechanical ventilation; PRISM = Pediatric Risk of Mortality Score; SA AKI = sepsis-associated acute kidney injury; SA ARD = sepsis-associated acute respiratory dysfunction.

a Other was categorized as any self-identified race other than Black or White.

b Adjusted for mortality prior to day 7.

**TABLE 2 ]:** Clinical Characteristics of Patients Classified as High-Risk and Low-Risk of Day 3 SA ARD in the Validation Data Set (n = 250)

Variable	Day 3 SA ARD		*P* Value

	High Risk (n = 156)	Low Risk (n = 94)	

Age, y	3.5 (1.1–7.9)	7.3 (2.9–12.1)	< .001
Sex, female	73 (46.8)	47 (50.4)	.567
Race (self-identified)			
White	117 (75.0)	67 (72.0)	.867
Black	20 (12.8)	10 (10.7)	
Other^a^	16 (12.2)	16 (17.2)	
Ethnicity (self-reported)			
Hispanic or Latino	4 (2.6)	0 (0)	
PRISM III score	11 (7–16)	10 (5–13)	.104
28-d Mortality	8 (5.3)	9 (9.6)	.176
Complicated course	54 (34.6)	16 (17.1)	.003
PICU LOS	8 (5–16)	2 (1–9)	< .001
PICU-free days	20 (11–23)	26 (19–27)	< .001
SA ARD			
Day 3	131 (85.1)	21 (30.0)	< .001
Day 7	68 (61.8)	11 (32.3)	.003
Mechanical ventilation			
Day 3	129 (83.8)	27 (39.2)	< .001
Day 7	71 (64.5)	16 (47.1)	.068
Pao_2_ to Fio_2_ ratio < 250			
Day 3	35 (22.8)	4 (5.8)	.002
Day 7	21 (19.1)	1 (3.0)	.022
SA AKI			
Day 3	51 (33.2)	21 (30.0)	.643
Day 7	38 (34.6)	13 (38.2)	.694
Any RRT	17 (10.9)	10 (10.8)	.972
CRRT			
Day 3	15 (9.6)	6 (6.5)	.385
Day 7	12 (7.7)	8 (8.6)	.798

Data are presented as No. (%) or median (interquartile range) unless otherwise indicated. CRRT = continuous renal replacement therapy; LOS = length of stay; PRISM = Pediatric Risk of Mortality Score; RRT = renal replacement therapy; SA AKI = sepsis-associated acute kidney injury; SA ARD = sepsis-associated acute respiratory dysfunction.

a Other was categorized as any self-identified race other than Black or White.

**TABLE 3 ]:** Diagnostic Test Characteristics of the Classification and Regression Tree Model Predictive of Sepsis-Associated Acute Respiratory Dysfunction on Day 3 in the Training, Validation, and Test Data Sets

Diagnostic Test Characteristic	Data (95% CI)

Training data set (n = 375)	
Sensitivity	0.91 (0.86–0.94)
Specificity	0.76 (0.68–0.82)
Positive predictive value	0.84 (0.78–0.88)
Negative predictive value	0.85 (0.78–0.91)
Positive likelihood ratio	3.73 (2.8–4.93)
Negative likelihood ratio	0.12 (0.08–0.18)
Validation data set (n = 250)	
Sensitivity	0.84 (0.77–0.90)
Specificity	0.73 (0.63–0.82)
Positive predictive value	0.84 (0.77–0.89)
Negative predictive value	0.74 (0.64–0.82)
Positive likelihood ratio	3.18 (2.26–4.47)
Negative likelihood ratio	0.21 (0.14–0.31)
Test data set (n = 162)	
Sensitivity	0.85 (0.80–0.91)
Specificity	0.64 (0.57–0.72)
Positive predictive value	0.64 (0.57–0.72)
Negative predictive value	0.85 (0.80–0.91)
Positive likelihood ratio	2.40 (1.79–3.22)
Negative likelihood ratio	0.23 (0.13–0.41)

## ABBREVIATIONS:

AUC: area under the receiver operating characteristic curve
CART: classification and regression tree
LOS: length of stay
OI: oxygenation index
SA ARD: sepsis-associated acute respiratory dysfunction
sTM: soluble thrombomodulin
VCAM-1: vascular cell adhesion molecule 1
