# Supplementary material for: Derivation and Validation of a Clinical and Endothelial Biomarker Risk Model to Predict Persistent Pediatric Sepsis-Associated Acute Respiratory Dysfunction
Source: CHEST Crit Care. Author manuscript; Available in PMC 2025 Apr 16. (PMC12001826; doi:10.1016/j.chstcc.2024.100120)
Supplement: MMC1 [file NIHMS2066685-supplement-MMC1.docx]

**Derivation and Validation of a Clinical and Endothelial Biomarker Risk Model to Predict Persistent Pediatric Sepsis-Associated Acute Respiratory Dysfunction.**

Authors: James. G. Williams, MD^1^; Jane E. Whitney, MD^2^; Scott L. Weiss, MD^3^; Brian M. Varisco, MD^1^; Nadir Yehya, MD^4^; and Mihir R. Atreya, MD, MPH^5^ for the Sepsis Genomics Collaborative and CHOP Sepsis Investigators.

Author Affiliations:

1. Division of Critical Care Medicine and Department of Pediatrics, University of Arkansas for Medical Sciences, Little Rock, AR, 72205, U.S.A.
2. Division of Medical Critical Care, Department of Pediatrics, Boston Children's Hospital, Harvard Medical School, Boston, MA 02115, U.S.A.
3. Division of Critical Care Medicine, Nemour’s Children’s Hospital, Wilmington, DE, 19803.
4. Division of Critical Care Medicine, Children's Hospital of Philadelphia, Philadelphia, PA 19104, U.S.A.
5. Division of Critical Care Medicine, Cincinnati Children's Hospital Medical Center and Department of Pediatrics University of Cincinnati, College of Medicine, Cincinnati, OH, 45229, U.S.A.

Corresponding author:

James G. Williams, MD,

Arkansas Children's Hospital

Division of Critical Care Medicine

13 Children's Way Slot 512-36

Little Rock, AR, 72202, U.S.A.

Tel: 501-364-7514

Email: [Jwilliams4@uams.edu](mailto:Jwilliams4@uams.edu)

**Table of contents:**

1. Supplemental Methods
2. Supplemental Tables.
3. Supplemental Figure Legend.
4. Supplemental Figures.

**Supplementary Methods:**

**Pre-existing Lung Disease:** Patients with a history of any of the 10 following co-morbid conditions were categorized as having pre-existing lung disease: (1) Severe Neurologic Injury with Tracheostomy and Ventilator Dependence, (2) Chronic Respiratory Failure, (3) Chronic Lung Disease (CLD), (4) Bronchopulmonary Dysplasia (BPD), (5) Restrictive lung disease, (6) Pulmonary hypertension, (7) Alveolar hypoventilation syndrome, (8) Cystic fibrosis, (9) Bronchiolitis obliterans and (10) Pulmonary hemosiderosis.

**Choice of biomarkers:** We chose a combination of previously measured biomarkers in the derivation and test cohorts. We evaluated Interleukin-8, a key pro-inflammatory chemokine that recruits neutrophils to sites of infection or injury, which is a critical component of the acute inflammatory response. IL-8 is particularly relevant in the context of lung injury and sepsis,^1^ where neutrophil-driven inflammation contributes to endothelial damage and increased vascular permeability. We evaluated Angpt-1, Angpt-2, and their common receptor Tie2, which together regulate the stability and permeability of the vascular endothelium.^2^ Angpt-2 counteracts Angpt-1 and destabilizes the endothelium, and increased concentrations of Angpt-2 are associated with increased odds of mortality in multiple pediatric cohorts.^3,4^  We also included several adhesion molecules, including ICAM-1, VCAM-1, and PECAM-1, that are involved in immune-cell migration to the site of injury,^5,6^ ICAM-1 has been associated with the severity of lung injury, length of mechanical ventilation, and mortality,^7,8^ while VCAM-1 has not been well studied in the pediatric population, but is associated with ventilator-induced lung injury in adults.^9^ Finally, we included soluble thrombomodulin, an anti-thrombotic protein expressed on the surface of the endothelium that is cleaved in response to endothelial injury and has been associated with increased mortality in pediatric cohorts.^10^

**References:**

1. Flori, H. *et al.* A prospective investigation of interleukin-8 levels in pediatric acute respiratory failure and acute respiratory distress syndrome. *Crit. Care Lond. Engl.* **23**, 128 (2019).

2. Augustin, H. G., Koh, G. Y., Thurston, G. & Alitalo, K. Control of vascular morphogenesis and homeostasis through the angiopoietin-Tie system. *Nat. Rev. Mol. Cell Biol.* **10**, 165–177 (2009).

3. Yehya, N. *et al.* Circulating markers of endothelial and alveolar epithelial dysfunction are associated with mortality in pediatric acute respiratory distress syndrome. *Intensive Care Med* **42**, 1137–45 (2016).

4. Zinter, M. S. *et al.* Plasma angiopoietin-2 outperforms other markers of endothelial injury in prognosticating pediatric ARDS mortality. *Am. J. Physiol. Lung Cell. Mol. Physiol.* **310**, L224-231 (2016).

5. Carlton, E. F. & Flori, H. R. Biomarkers in pediatric acute respiratory distress syndrome. *Ann. Transl. Med.* **7**, 505 (2019).

6. Joffre, J., Hellman, J., Ince, C. & Ait-Oufella, H. Endothelial Responses in Sepsis. *Am. J. Respir. Crit. Care Med.* **202**, 361–370 (2020).

7. Williams, J. G. *et al.* Comparison of 16 Pediatric Acute Respiratory Distress Syndrome-Associated Plasma Biomarkers With Changing Lung Injury Severity. *Pediatr. Crit. Care Med. J. Soc. Crit. Care Med. World Fed. Pediatr. Intensive Crit. Care Soc.* **25**, e31–e40 (2024).

8. Flori, H. R., Ware, L. B., Glidden, D. & Matthay, M. A. Early elevation of plasma soluble intercellular adhesion molecule-1 in pediatric acute lung injury identifies patients at increased risk of death and prolonged mechanical ventilation. *Pediatr. Crit. Care Med. J. Soc. Crit. Care Med. World Fed. Pediatr. Intensive Crit. Care Soc.* **4**, 315–321 (2003).

9. Attia, E. F., Jolley, S. E., Crothers, K., Schnapp, L. M. & Liles, W. C. Soluble Vascular Cell Adhesion Molecule-1 (sVCAM-1) Is Elevated in Bronchoalveolar Lavage Fluid of Patients with Acute Respiratory Distress Syndrome. *PLoS ONE* **11**, e0149687 (2016).

10. Monteiro, A. C. C. *et al.* Thrombomodulin is associated with increased mortality and organ failure in mechanically ventilated children with acute respiratory failure: biomarker analysis from a multicenter randomized controlled trial. *Crit. Care Lond. Engl.* **25**, 271 (2021).

**e-Table 1.** Univariate associations between clinical and endothelial biomarker variables tested and presence of sepsis associated acute respiratory dysfunction on day 3 (D3 SA-ARD) in the training dataset (n=375).

|  | Odds ratio | P value |
| --- | --- | --- |
| **Clinical variables** |  |  |
| Age | 0.92 (0.89, 0.95) | <0.001 |
| PRISM-III | 1.05 (1.02) | <0.001 |
| Pre-existing Comorbidity | 0.52 (0.33, 0.77) | 0.002 |
| Immunocompromised | 0.49 (0.29, 0.83) | 0.008 |
| P/F Ratio < 250 | 16.1 (6.3, 40.8) | <0.001 |
| D1 SA-ARD | 32.0 (16.8, 60.5) | <0.001 |
|  |  |  |
| **Endothelial biomarkers** |  |  |
| Angpt-1 (log10) | 0.89 (0.57, 1.39) | 0.606 |
| Angpt-2 (log10) | 2.75 (1.65, 4.58) | <0.001 |
| Tie-2 (log10) | 0.99 (0.47, 2.06) | 0.973 |
| Angpt-2/Angpt-1 ratio | 1.07 (1.01, 1.13) | 0.017 |
| Angpt-2/Tie-2 ratio | 1.18 (1.02, 1.36) | 0.021 |
| IL-8 (log10) | 1.74 (1.32, 2.29) | <0.001 |
| sTM (log10) | 4.41 (1.80, 10.8) | 0.001 |
| PECAM-1 (log10) | 3.42 (1.37, 8.54) | 0.009 |
| ICAM-1 (log10) | 2.93 (1.18, 7.25) | 0.020 |
| VCAM-1 (log10) | 0.40 (0.18, 0.88) | 0.022 |

**e-Table 2.** Biomarkers selected based on multivariate associations with backward elimination between and presence of sepsis associated acute respiratory dysfunction on day 3 (D3 SA-ARD) in the training dataset (n=375).

|  | Odds ratio | P value |
| --- | --- | --- |
| **Endothelial biomarkers** |  |  |
| Angpt-1 (log10) | 0.41 (0.20, 0.84) | 0.016 |
| VCAM-1 (log10) | 0.18 (0.05, 0.61) | 0.005 |
| sTM (log10) | 5.98 (1.5, 23.7) | 0.011 |

All clinical variables were coerced into the model.

Alpha of 0.1 used for backward elimination of biomarkers.

**e-Table 3:** Model summary of TreeNet® Model inclusive of only clinical variables including (1) age, (2) PRISM-III score, (3) pre-existing comorbidity, (4) history of immunocompromised status, (5) presence of sepsis associated acute respiratory dysfunction on day 1 (D1 SA-ARD), and (6) PaO2/FiO2 ratio < 250 to predict risk of day 3 sepsis associated acute respiratory dysfunction (D3 SA-ARD) in the training dataset (n=375).

| Total predictors | 6 | |
| --- | --- | --- |
| Important predictors | 5 | |
| Number of trees grown | 300 | |
| Optimal number of trees | 37 | |
| Statistics | Training | Test |
| Average -loglikelihood | 0.5594 | 0.5735 |
| Area under ROC curve | 0.9151 | 0.8766 |
| 95% CI | (0.8868, 0.9434) | (0.8389, 0.9143) |
| Lift | 2.0000 | 1.9364 |
| Weighted misclassification rate | 0.1858 | 0.1872 |

**e-Table 4:** Confusion Matrix of TreeNet® Model inclusive of only clinical variables including (1) age, (2) PRISM-III score, (3) pre-existing comorbidity, (4) history of immunocompromised status, (5) presence of sepsis associated acute respiratory dysfunction on day 1 (D1 SA-ARD), and (6) PaO2/FiO2 ratio < 250 to predict risk of day 3 sepsis associated acute respiratory dysfunction (D3 SA-ARD) in the training dataset (n=375).

|  |  | Predicted Class (Training) | | | Predicted Class (Test) | | |
| --- | --- | --- | --- | --- | --- | --- | --- |
|  |  |  |  |  |  |  |  |
| Actual Class | Count | 1.00 | 0.00 | % Correct | 1.00 | 0.00 | % Correct |
| 1.00 (Event) | 219 | 205 | 14 | 93.61 | 203 | 16 | 92.69 |
| 0.00 | 156 | 48 | 108 | 69.23 | 47 | 109 | 69.87 |
| All | 375 | 253 | 122 | 83.47 | 250 | 125 | 83.20 |

| Statistics | Training (%) | Test (%) |
| --- | --- | --- |
| True positive rate (sensitivity or power) | 93.61 | 92.69 |
| False positive rate (type I error) | 30.77 | 30.13 |
| False negative rate (type II error) | 6.39 | 7.31 |
| True negative rate (specificity) | 69.23 | 69.87 |

**e-Table 5:** Model summary of TreeNet® Model inclusive of all clinical variables and selected endothelial biomarkers with independent association with outcome of interest to predictive of day 3 sepsis associated acute respiratory dysfunction (D3 SA-ARD) in the training dataset (n=375).

| Total predictors | 9 | |
| --- | --- | --- |
| Important predictors | 8 | |
| Number of trees grown | 300 | |
| Optimal number of trees | 146 | |
| Statistics | Training | Test |
| Average -loglikelihood | 0.3897 | 0.4573 |
| Area under ROC curve | 0.9605 | 0.8762 |
| 95% CI | (0.9434, 0.9776) | (0.8384, 0.9139) |
| Lift | 2.0000 | 2.0017 |
| Weighted misclassification rate | 0.1657 | 0.1776 |

**e-Table 6:** Confusion Matrix of TreeNet® Model inclusive of all clinical variables and selected endothelial biomarkers with independent association with outcome of interest to predictive of day 3 sepsis associated acute respiratory dysfunction (D3 SA-ARD) in the training dataset (n=375).

|  | | Predicted Class (Training) | | | Predicted Class (Test) | | |
| --- | --- | --- | --- | --- | --- | --- | --- |
| Actual Class | Count | 1.00 | 0.00 | % Correct | 1.00 | 0.00 | % Correct |
| 1.00 (Event) | 219 | 204 | 15 | 93.15 | 203 | 16 | 92.69 |
| 0.00 | 156 | 41 | 115 | 73.72 | 44 | 112 | 71.79 |
| All | 375 | 245 | 130 | 85.07 | 247 | 128 | 84.00 |

| Statistics | Training (%) | Test (%) |
| --- | --- | --- |
| True positive rate (sensitivity or power) | 93.15 | 92.69 |
| False positive rate (type I error) | 26.28 | 28.21 |
| False negative rate (type II error) | 6.85 | 7.31 |
| True negative rate (specificity) | 73.72 | 71.79 |

| Variable | High D3 SA-ARD risk (n=156) | Low D3 SA-ARD risk (n=93) | p value |
| --- | --- | --- | --- |
|  |  |  |  |
| Age (Years) | 3.5 (1.1, 7.9) | 7.3 (2.9, 12.1) | <0.001 |
| Sex, Female (%) | 73 (46.8) | 47 (50.4%) | 0.567 |
| Race (Self-identified) |  |  |  |
| White/Caucasian | 117 (75.0%) | 67 (72.0%) | 0.867 |
| Black/African American | 20 (12.8%) | 10 (10.7%) |  |
| Other | 16 (12.2%) | 16 (17.2%) |  |
| Ethnicity |  |  |  |
| Hispanic or Latino | 4 (2.6%) | 0 (0) |  |
| PRISM-III | 11 (7, 16) | 10 (5, 13) | 0.104 |
|  |  |  |  |
| 28-day Mortality | 8 (5.3%) | 9 (9.6%) | 0.176 |
| Complicated course | 54 (34.6%) | 16 (17.1%) | 0.003 |
| PICU LOS | 8 (5, 16) | 2 (1, 9) | <0.001 |
| PICU Free Days | 20 (11, 23) | 26 (19, 27) | <0.001 |
| D3 SA-ARD | 131 (85.1%) | 21 (30.0%) | <0.001 |
| D7 SA-ARD | 68 (61.8 %) | 11 (32.3%) | 0.003 |
| D3 MV | 129 (83.8%) | 27 (39.2%) | <0.001 |
| D7 MV | 71 (64.5%) | 16 (47.1%) | 0.068 |
| D3 P/F <250 | 35 (22.8%) | 4 (5.8%) | 0.002 |
| D7 P/F <250 | 21 (19.1%) | 1 (3.0%) | 0.022 |
| D3 SA-AKI | 51 (33.2%) | 21 (30.0%) | 0.643 |
| D7 SA-AKI | 38 (34.6%) | 13 (38.2%) | 0.694 |
| Any CRRT | 17 (10.9%) | 10 (10.8%) | 0.972 |
| D3 CRRT | 15 (9.6%) | 6 (6.5%) | 0.385 |
| D7 CRRT | 12 (7.7%) | 8 (8.6%) | 0.798 |

**e-Table 7.** Clinical characteristics of patients classified as high- and low-risk of day 3 sepsis associated acute respiratory dysfunction (D3 SA-ARD) in the hold-out validation dataset (n=250).

PRISM III: Pediatric risk of mortality score III

LOS: Length of stay

SA-ARD: Sepsis associated acute respiratory dysfunction.

MV: Mechanical ventilation

P/F: PaO2/FiO2 ratio.

SA-AKI: Sepsis Associated Acute Kidney Injury.

CRRT: Continuous Renal Replacement Therapy.

**e-Table 8.** Comparison of patients with and without persistent sepsis associated acute respiratory dysfunction at in the test cohort (n=162).

| Variable | D3 SA-ARD (n=71) | D3 SA-ARD (n=91) | P value |
| --- | --- | --- | --- |
|  |  |  |  |
| Age (Years) | 9.9 (2.9, 14.1) | 6.8 (3.3, 14.5) | 0.552 |
| Sex, Female (%) | 31 (43.7%) | 49 (53.9%) | 0.198 |
| Race (Self-identified) |  |  | 0.602 |
| White/Caucasian | 35 (49.3%) | 46 (50.6%) |  |
| Black/African American | 16 (22.5%) | 25 (27.5%) |  |
| Other | 20 (28.2%) | 20 (22.0%) |  |
| Ethnicity |  |  | 0.865 |
| Hispanic or Latino | 11 (15.5%) | 11 (16.5%) |  |
| PRISM-III | 14 (7, 19) | 10 (5,15) | 0.013 |
|  |  |  |  |
| PICU Mortality | 11 (15.5%) | 8 (8.8%) | 0.188 |
| Complicated course | 67 (94.4%) | 46 (50.6%) | <0.001 |
| PICU LOS | 15 (9, 28) | 7 (3, 16) | <0.001 |
| PICU Free Days | 13 (0, 19) | 21 (12, 25) | <0.001 |
| Day 7 MV | 71 (100%) | 24 (42.1) | <0.001 |
| Day 7 Intubation | 71 (100%) | 24 (42.1) | <0.001 |
| Day 7 P/F <250 | 28 (43.1%) | 1 (5.9) | 0.004 |
| Day 7 CRRT* | 2 (20%) | 0 (0%) | 0.092 |
| Day 1-7 %PFO | 11.5% (5.7, 18.5) | 8.5% (3.4, 20.0) | 0.179 |
| Day 1-7 Cardiac Arrest | N/A | N/A |  |
| Positive blood culture | 15 (21.1%) | 28 (30.8%) | 0.168 |
| Positive culture | 51 (71.8%) | 63 (69.2%) | 0.719 |
| Source |  |  | 0.255 |
| Pulmonary | 24 (47.1%) | 23 (36.5%) |  |
| Extra-pulmonary | 27 (52.9%) | 40 (63.5%) |  |
| Steroids | 37 (57.8%) | 39 (70.9%) | 0.138 |

**e-Figure legend:**

**e-Figure 1.** Flow diagram demonstrating inclusion and exclusion of patients in the derivation cohort.

**e-Figure 2.** Relative variable importance shown in TreeNet® models inclusive only of clinical variables.

**e-Figure 3.** Relative variable importance shown in TreeNet® models inclusive of clinical variables and select endothelial biomarkers.

**e-Figure 4.** Area under the receiver operating characteristic curve (AUROC) and weighted misclassification chart for TreeNet® Model inclusive of clinical variables and select endothelial biomarkers predictive of day 3 sepsis associated acute respiratory dysfunction (D3 SA-ARD) in the training dataset (n=375).

**e-Figure 5.** Predictor partial dependence plots between concentrations in pg/mL of select endothelial biomarkers sTM, VCAM-1, and Angpt-1 on the x-axis and fitted half log odds of D3 SA-ARD, the latter shown on the y-axis.

**e-Figure 6.** Surface plot showing interaction between soluble thrombomodulin (sTM) and vascular cell adhesion molecule-1 (VCAM-1) on log odds of persistent sepsis associated acute respiratory dysfunction (D3 SA-ARD) among patients in the training dataset. Patients with high sTM and VCAM-1 concentrations had higher odds of D3 SA-ARD. However, a relatively small subset of patients with low sTM concentrations and had higher VCAM-1 concentrations had a lower odds of D3 SA-ARD suggestive of an interaction between these biomarkers.

**e-Figure 7.** Area under the receiver operating characteristic curve for Classification and

Regression Tree (CART) model predictive of day 3 sepsis associated acute respiratory dysfunction (D3 SA-ARD) in hold-out validation dataset (n=250).

**e-Figure 8.** Classification and regression tree (CART) showing number of patients with and without sepsis associated acute respiratory dysfunction at timepoint 2 (T2 SA-ARD) according to terminal nodes in the test cohort (n=162).

**e-Figure 9.** Area under the receiver operating characteristic curve (AUROC) of classification and regression tree (CART) model predictive of persistent sepsis associated acute respiratory dysfunction at time point 2 (T2 SA-ARD) the test cohort (n=162).
