# Supplementary material for: Derivation and Validation of a Clinical and Endothelial Biomarker Risk Model to Predict Persistent Pediatric Sepsis-Associated Acute Respiratory Dysfunction
Source: CHEST Crit Care. Author manuscript; Available in PMC 2025 Apr 16. (PMC12001826; doi:10.1016/j.chstcc.2024.100120)
Supplement: MMC2 [file NIHMS2066685-supplement-MMC2.docx]

**Table 1:** Presence of D3 Sepsis Associated Respiratory Dysfunction (D3 SA-ARD) comparing patients enrolled between 2002-2013 and 2013-2023 in training dataset.

| D3 SA-ARD | 2002-2013 | 2013-2023 | p value |
| --- | --- | --- | --- |
| No | 18 (58.1%) | 138 (40.1%) | 0.052 |
| Yes | 13 (41.9%) | 206 (59.9%) |  |
|  | 31 | 344 |  |

**Table 2.** Biomarkers selected based on multivariate associations with backward elimination between and presence of sepsis associated acute respiratory dysfunction on day 3 (D3 SA-ARD) in the training dataset only including patients recruited between 2013-2023 (n=344).

|  | Odds ratio | P value |
| --- | --- | --- |
| **Endothelial biomarkers** |  |  |
| Angpt-1 (log10) | 0.36 (0.15, 0.83) | 0.017 |
| VCAM-1 (log10) | 0.14 (0.03, 0.54) | 0.004 |
| sTM (log10) | 16.11 (3.09, 83.91) | 0.001 |

All clinical variables were coerced into the model.

Alpha of 0.1 used for backward elimination of biomarkers.

**Table 3** Model summary of TreeNet® Model inclusive of only clinical variables including (1) age, (2) PRISM-III score, (3) pre-existing comorbidity, (4) history of immunocompromised status, (5) presence of sepsis associated acute respiratory dysfunction on day 1 (D1 SA-ARD), and (6) PaO2/FiO2 ratio < 250 to predict risk of day 3 sepsis associated acute respiratory dysfunction (D3 SA-ARD) in the training dataset only including patients recruited between 2013-2023 (n=344).

| Total predictors | 6 | |
| --- | --- | --- |
| Important predictors | 6 | |
| Number of trees grown | 300 | |
| Optimal number of trees | 67 | |
| Statistics | **Training** | **Test** |
| Average -loglikelihood | 0.4506 | 0.4706 |
| Area under ROC curve | 0.9465 | 0.8944 |
| 95% CI | (0.9250, 0.9680) | (0.8592, 0.9295) |
| Lift | 1.6699 | 1.6710 |
| Weighted misclassification rate | 0.1308 | 0.1396 |

**Table 4:** Confusion Matrix of TreeNet® Model inclusive of only clinical variables including (1) age, (2) PRISM-III score, (3) pre-existing comorbidity, (4) history of immunocompromised status, (5) presence of sepsis associated acute respiratory dysfunction on day 1 (D1 SA-ARD), and (6) PaO2/FiO2 ratio < 250 to predict risk of day 3 sepsis associated acute respiratory dysfunction (D3 SA-ARD) in the training dataset only including patients recruited between 2013-2023 (n=344).

|  |  | Predicted Class (Training) | | | Predicted Class (Test) | | |
| --- | --- | --- | --- | --- | --- | --- | --- |
|  |  |  |  |  |  |  |  |
| Actual Class | Count | 1.00 | 0.00 | % Correct | 1.00 | 0.00 | % Correct |
| 1.00 (Event) | 206 | 195 | 11 | 94.66 | 193 | 13 | 93.69 |
| 0.00 | 138 | 34 | 104 | 75.36 | 35 | 103 | 74.64 |
| All | 344 | 229 | 115 | 86.92 | 228 | 116 | 86.05 |

| Statistics | Training (%) | Test (%) |
| --- | --- | --- |
| True positive rate (sensitivity or power) | 94.66 | 93.69 |
| False positive rate (type I error) | 24.64 | 25.36 |
| False negative rate (type II error) | 5.34 | 6.31 |
| True negative rate (specificity) | 75.36 | 74.64 |

**Table 5** Model summary of TreeNet® Model inclusive of all clinical variables and selected endothelial biomarkers with independent association with outcome of interest to predictive of day 3 sepsis associated acute respiratory dysfunction (D3 SA-ARD) in the training dataset only including patients recruited between 2013-2023 (n=344).

| Total predictors | 9 | |
| --- | --- | --- |
| Important predictors | 8 | |
| Number of trees grown | 300 | |
| Optimal number of trees | 93 | |
| Statistics | Training | Test |
| Average -loglikelihood | 0.3965 | 0.4397 |
| Area under ROC curve | 0.9582 | 0.8908 |
| 95% CI | (0.9385, 0.9779) | (0.8523, 0.9294) |
| Lift | 1.6699 | 1.6010 |
| Weighted misclassification rate | 0.1308 | 0.1396 |

**Table 6:** Confusion Matrix of TreeNet® Model inclusive of all clinical variables and selected endothelial biomarkers with independent association with outcome of interest to predictive of day 3 sepsis associated acute respiratory dysfunction (D3 SA-ARD) in the training dataset only including patients recruited between 2013-2023 (n=344).

|  | | Predicted Class (Training) | | | Predicted Class (Test) | | |
| --- | --- | --- | --- | --- | --- | --- | --- |
| Actual Class | Count | 1.00 | 0.00 | % Correct | 1.00 | 0.00 | % Correct |
| 1.00 (Event) | 206 | 195 | 11 | 94.66 | 193 | 13 | 93.69 |
| 0.00 | 138 | 34 | 104 | 75.36 | 35 | 103 | 74.64 |
| All | 344 | 229 | 115 | 86.92 | 228 | 116 | 86.05 |

| Statistics | Training (%) | Test (%) |
| --- | --- | --- |
| True positive rate (sensitivity or power) | 94.66 | 93.69 |
| False positive rate (type I error) | 24.64 | 25.36 |
| False negative rate (type II error) | 5.34 | 6.31 |
| True negative rate (specificity) | 75.36 | 74.64 |
