# Supplementary figures and images for: Derivation and Validation of a Clinical and Endothelial Biomarker Risk Model to Predict Persistent Pediatric Sepsis-Associated Acute Respiratory Dysfunction
Source: CHEST Crit Care. Author manuscript; Available in PMC 2025 Apr 16. (PMC12001826; doi:10.1016/j.chstcc.2024.100120)

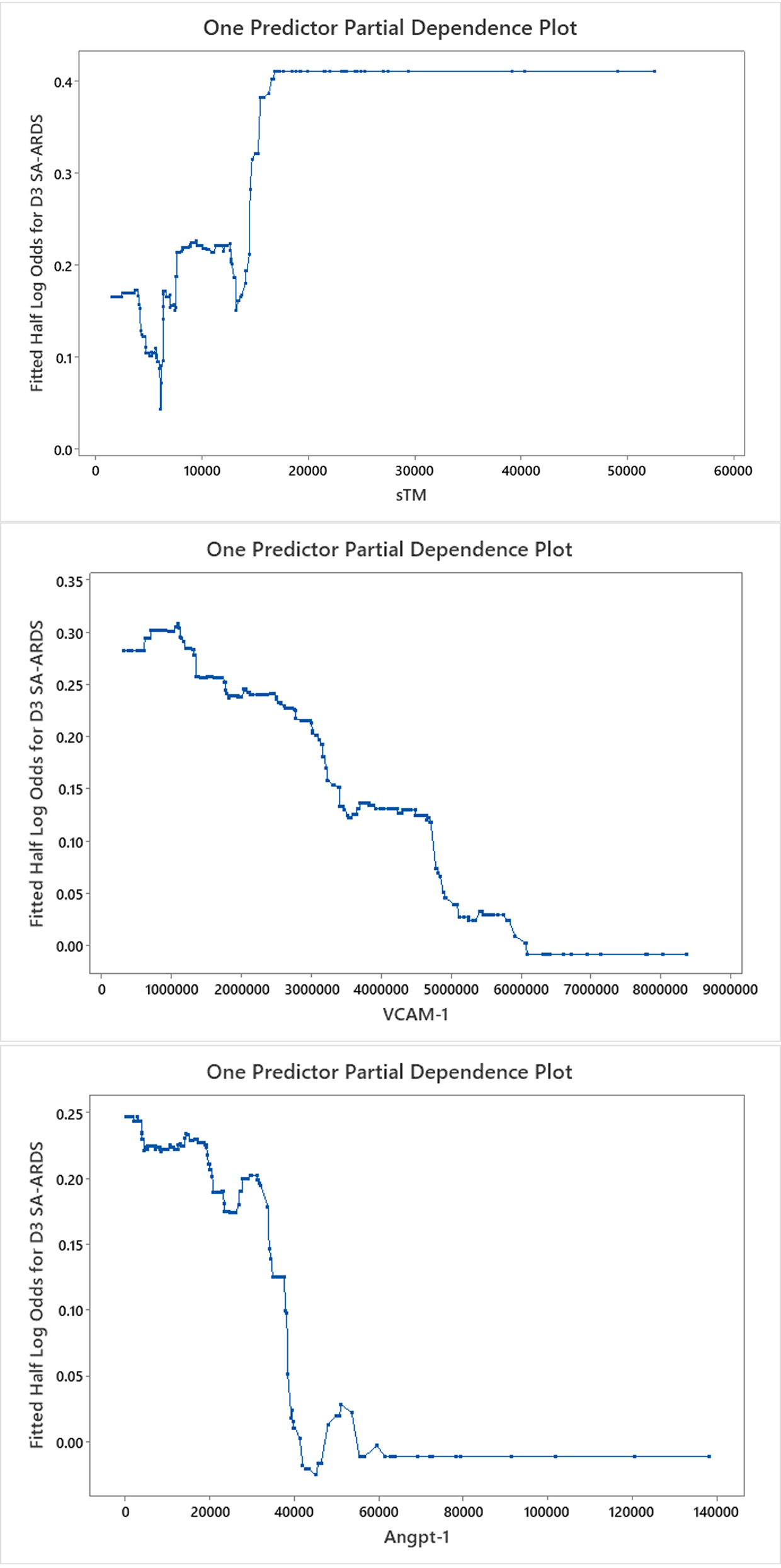

Supplement: fig5 [file NIHMS2066685-supplement-fig5.jpg]

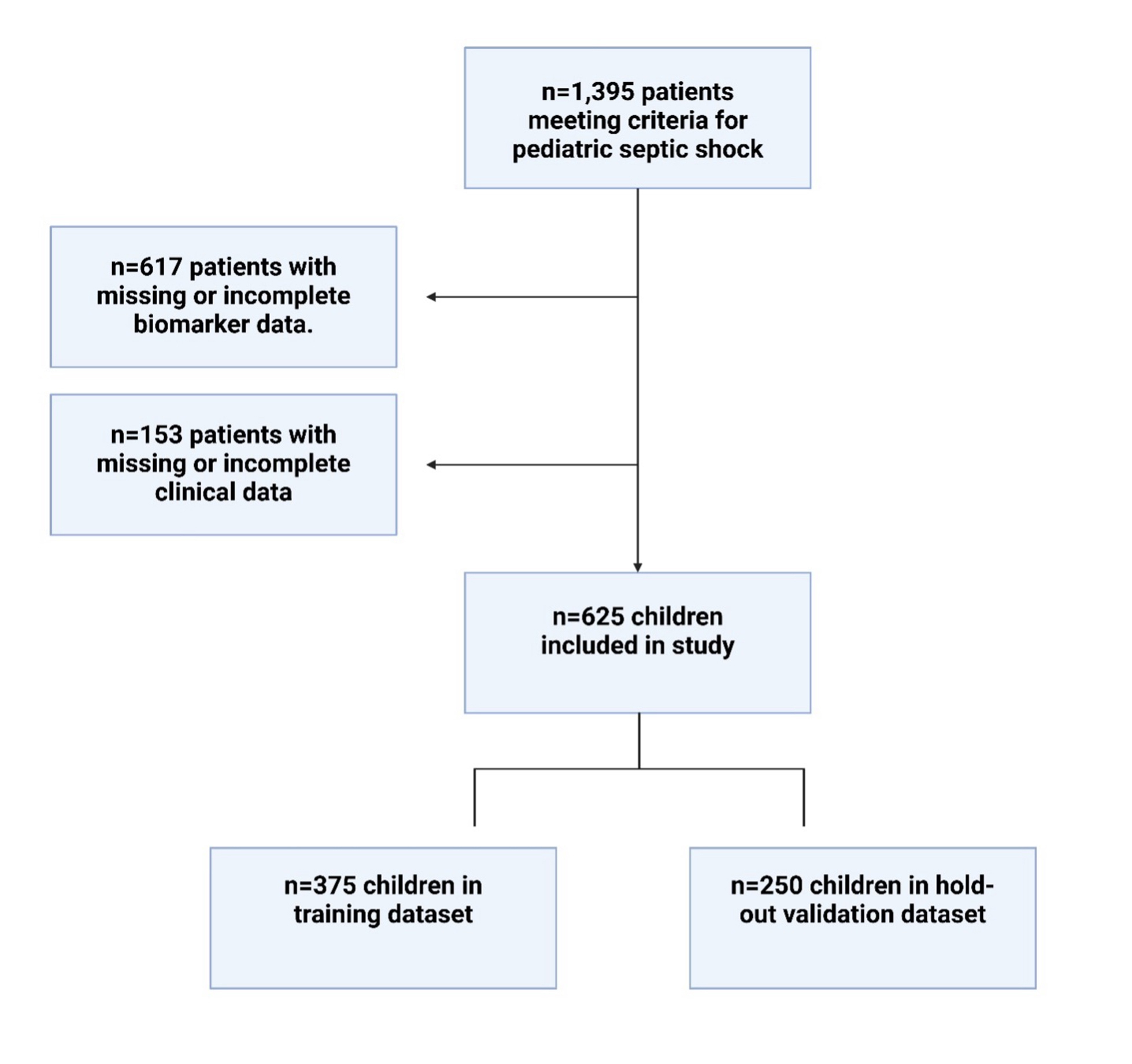

Supplement: fig1 [file NIHMS2066685-supplement-fig1.jpg]

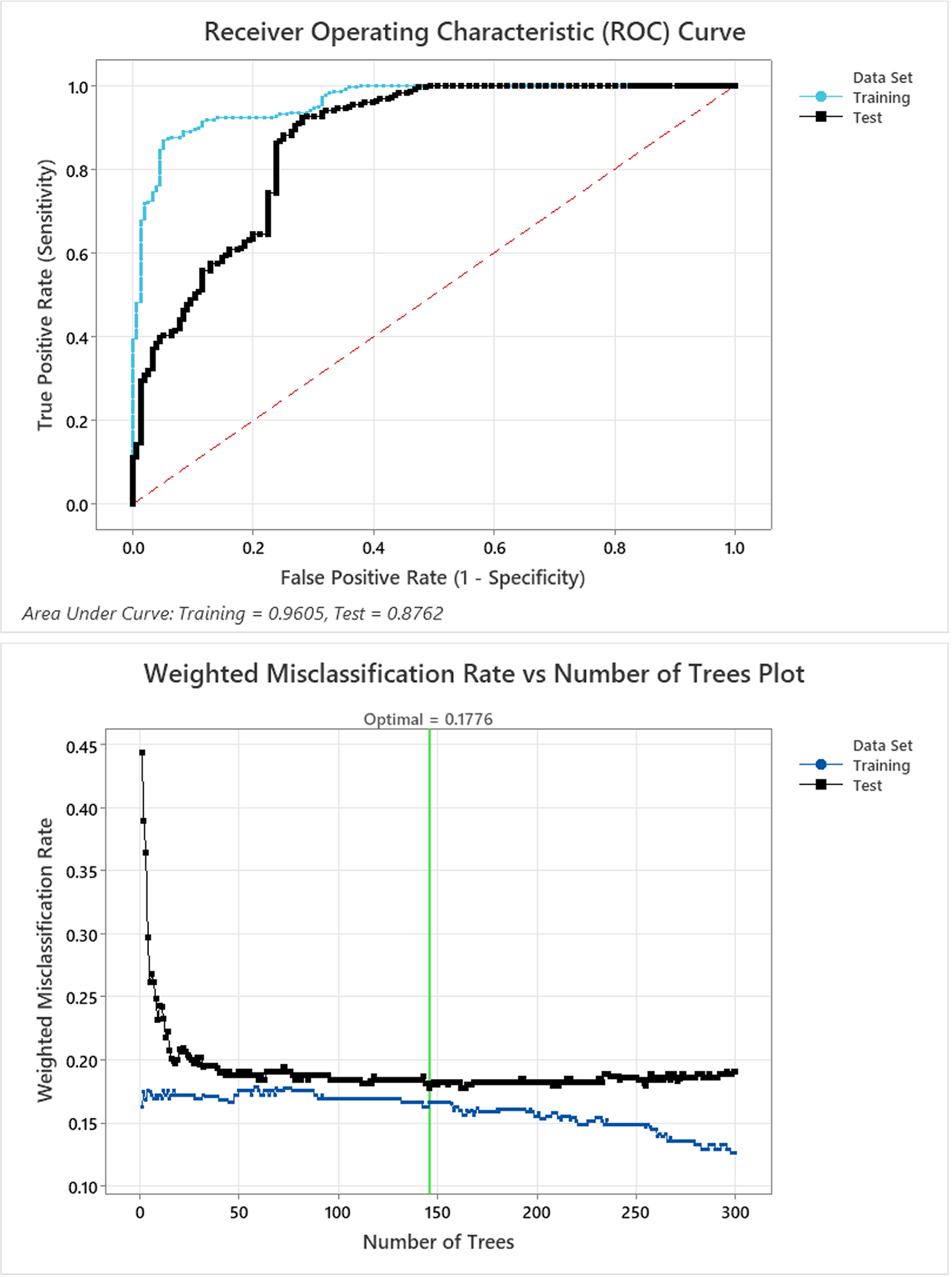

Supplement: fig4 [file NIHMS2066685-supplement-fig4.jpg]

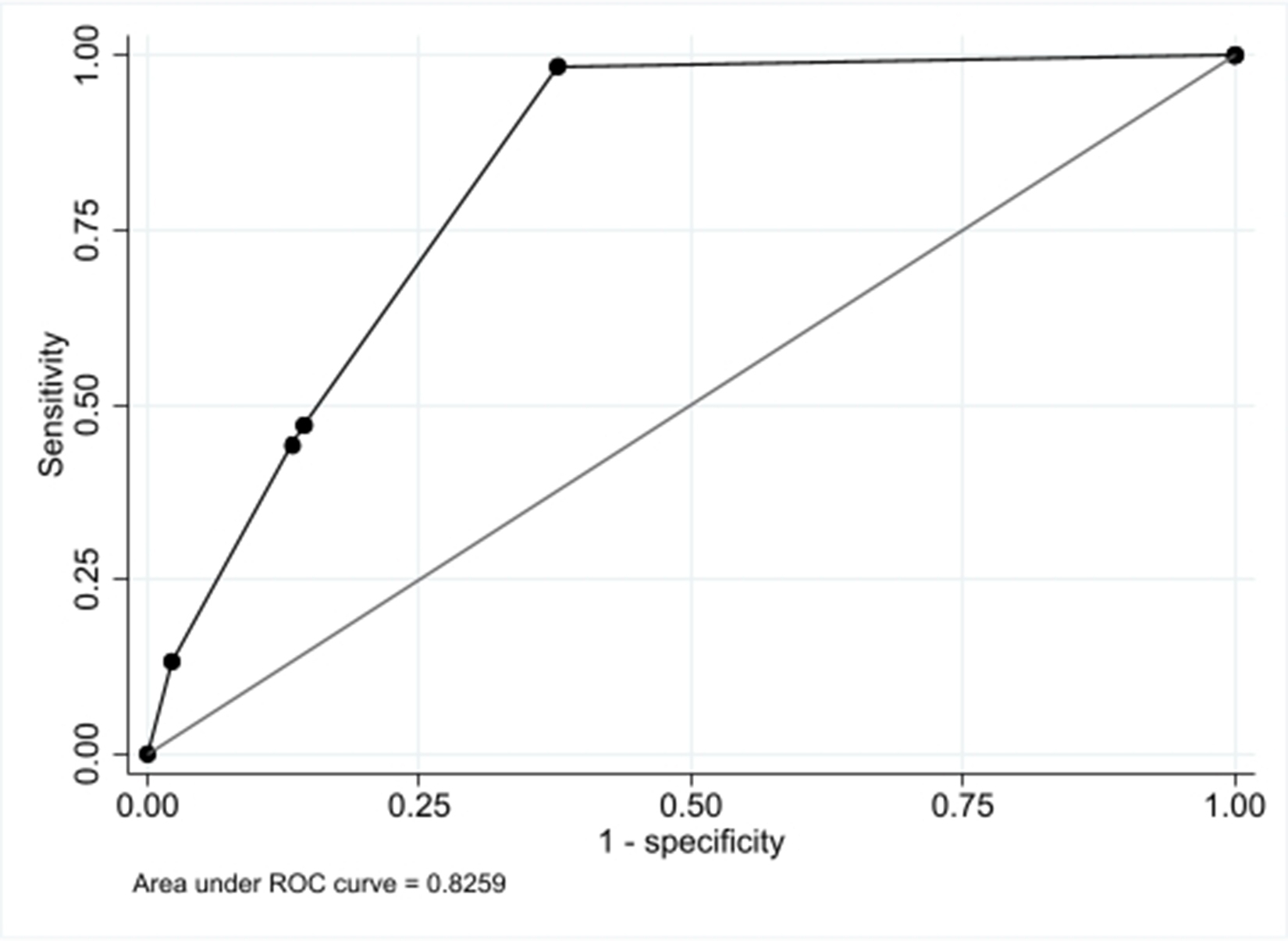

Supplement: fig9 [file NIHMS2066685-supplement-fig9.jpg]

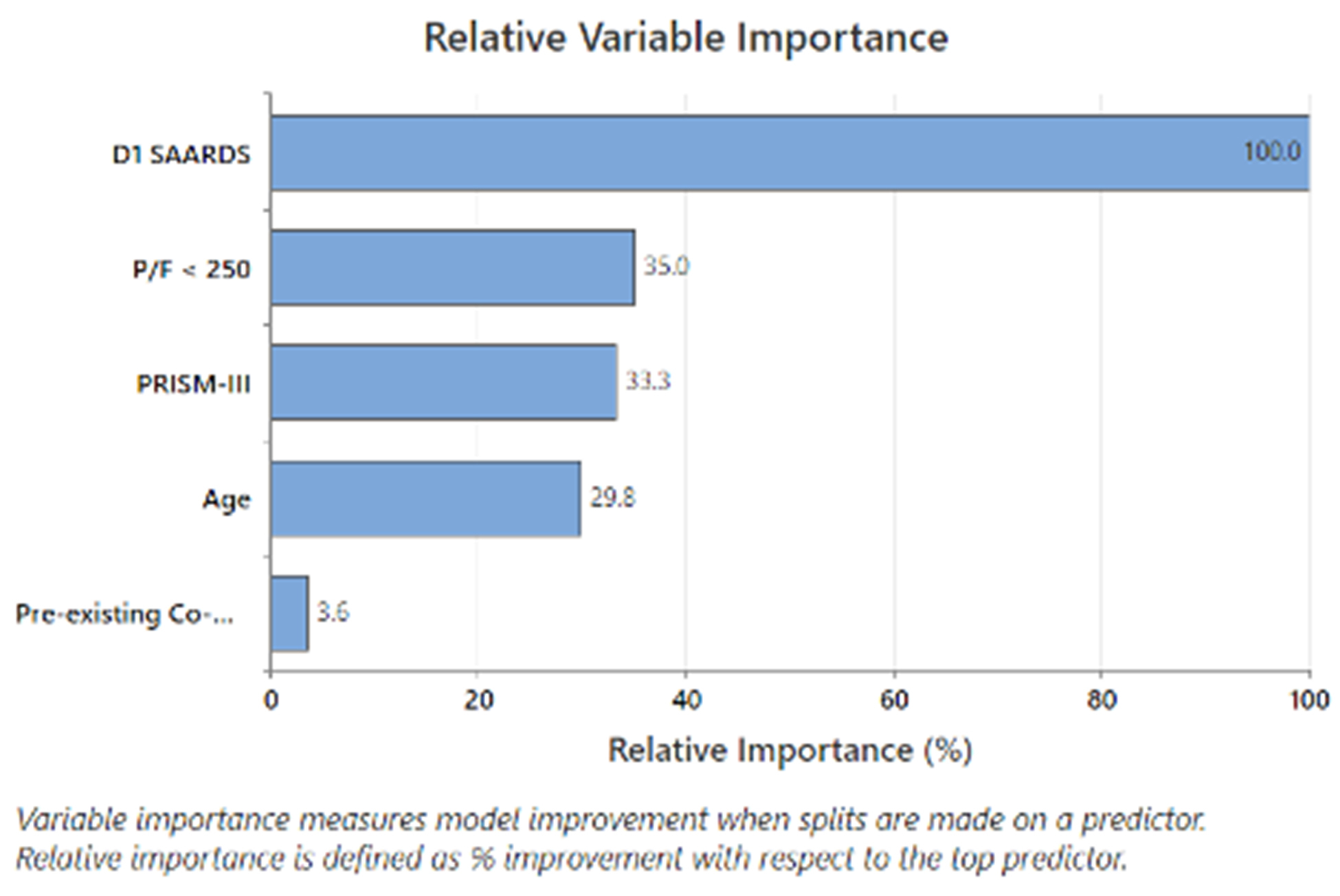

Supplement: fig2 [file NIHMS2066685-supplement-fig2.jpg]

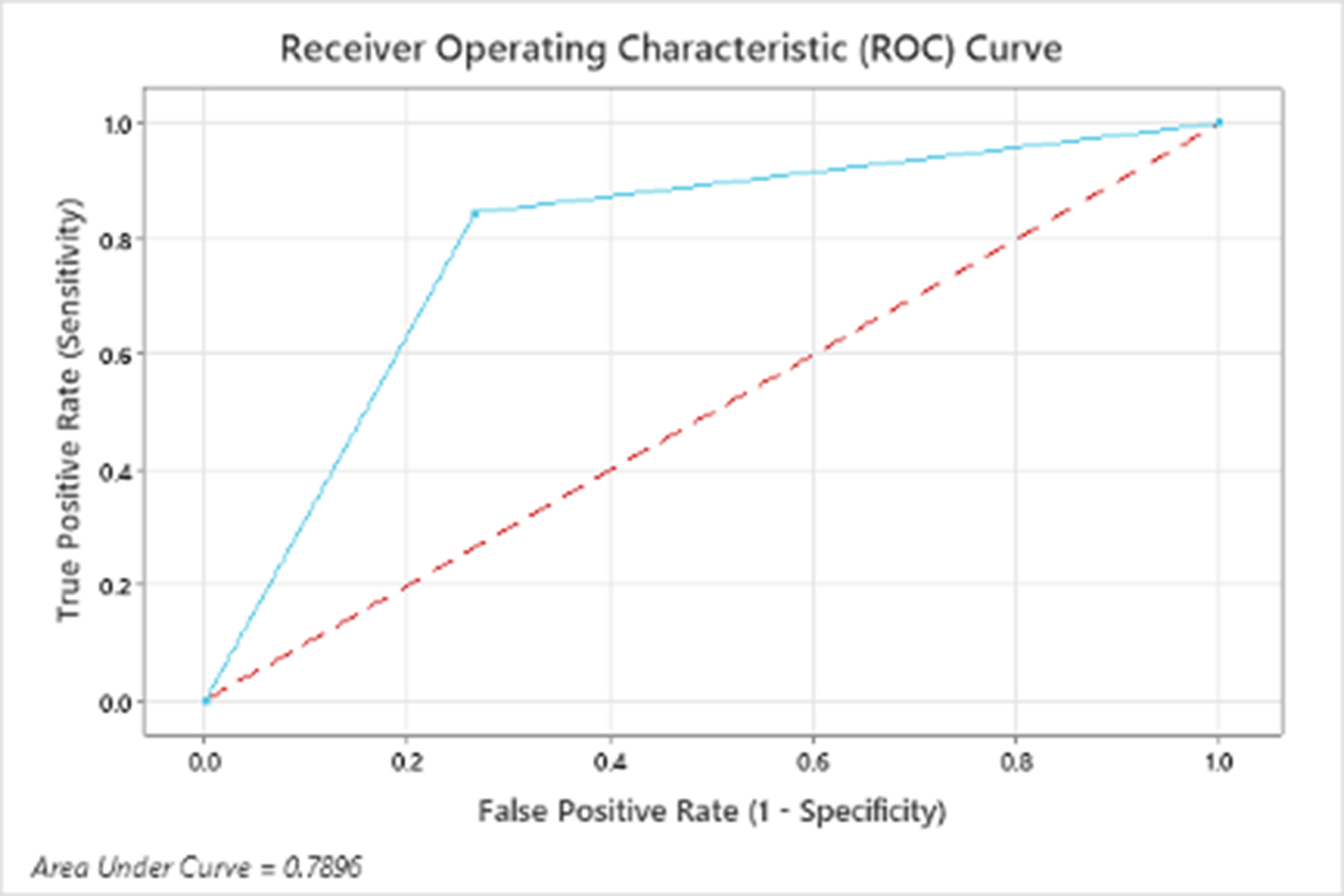

Supplement: fig7 [file NIHMS2066685-supplement-fig7.jpg]

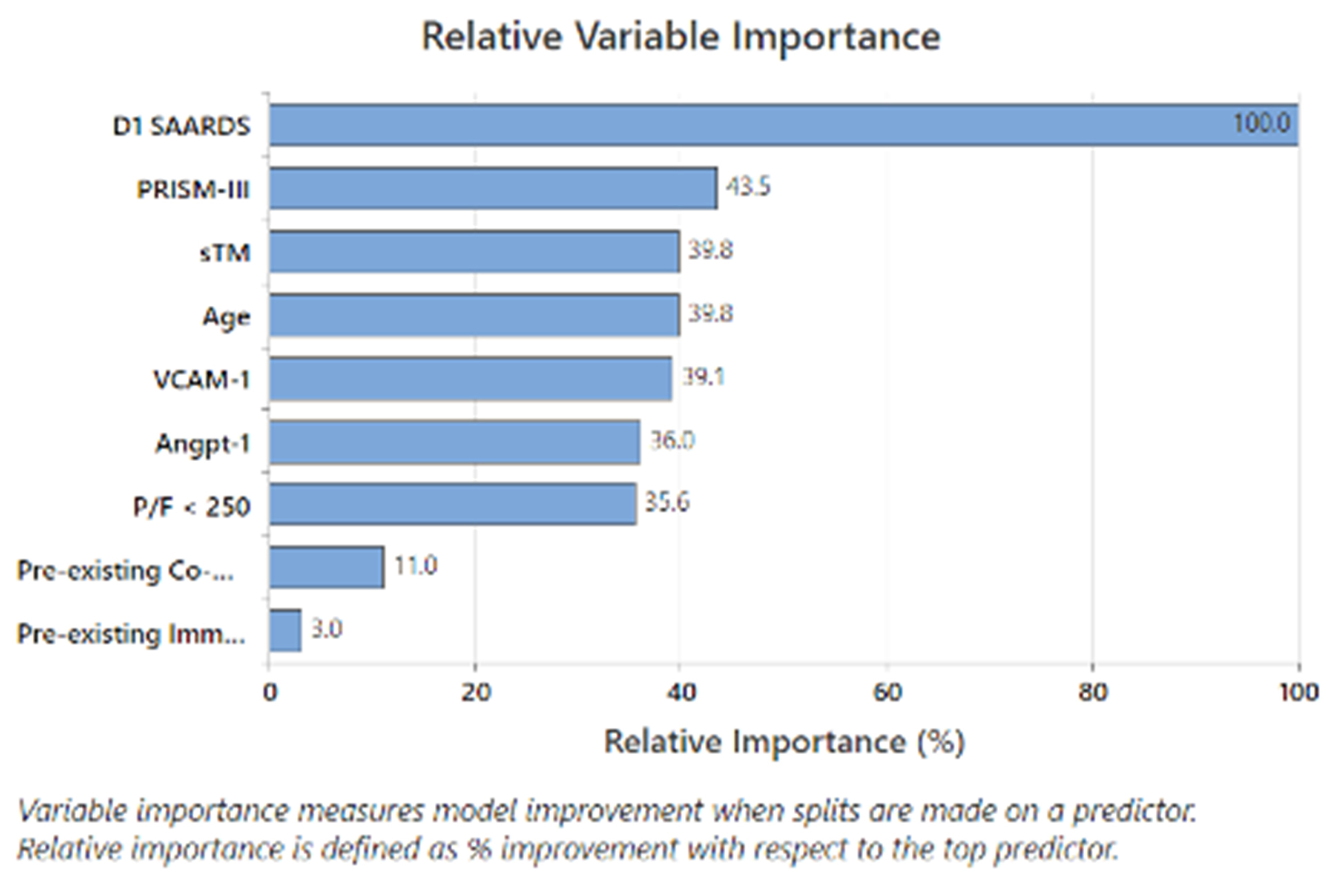

Supplement: fig3 [file NIHMS2066685-supplement-fig3.jpg]

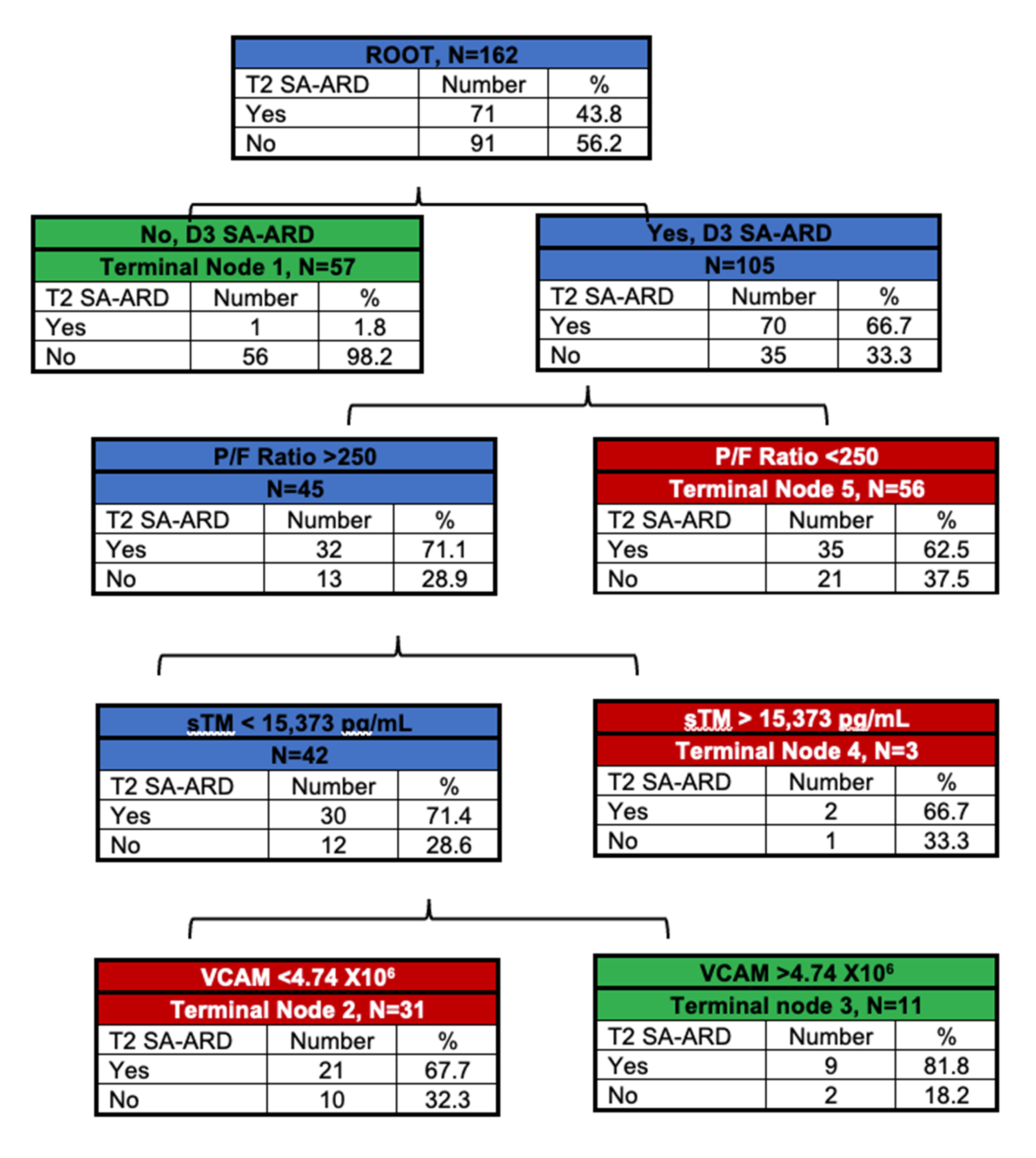

Supplement: fig8 [file NIHMS2066685-supplement-fig8.jpg]

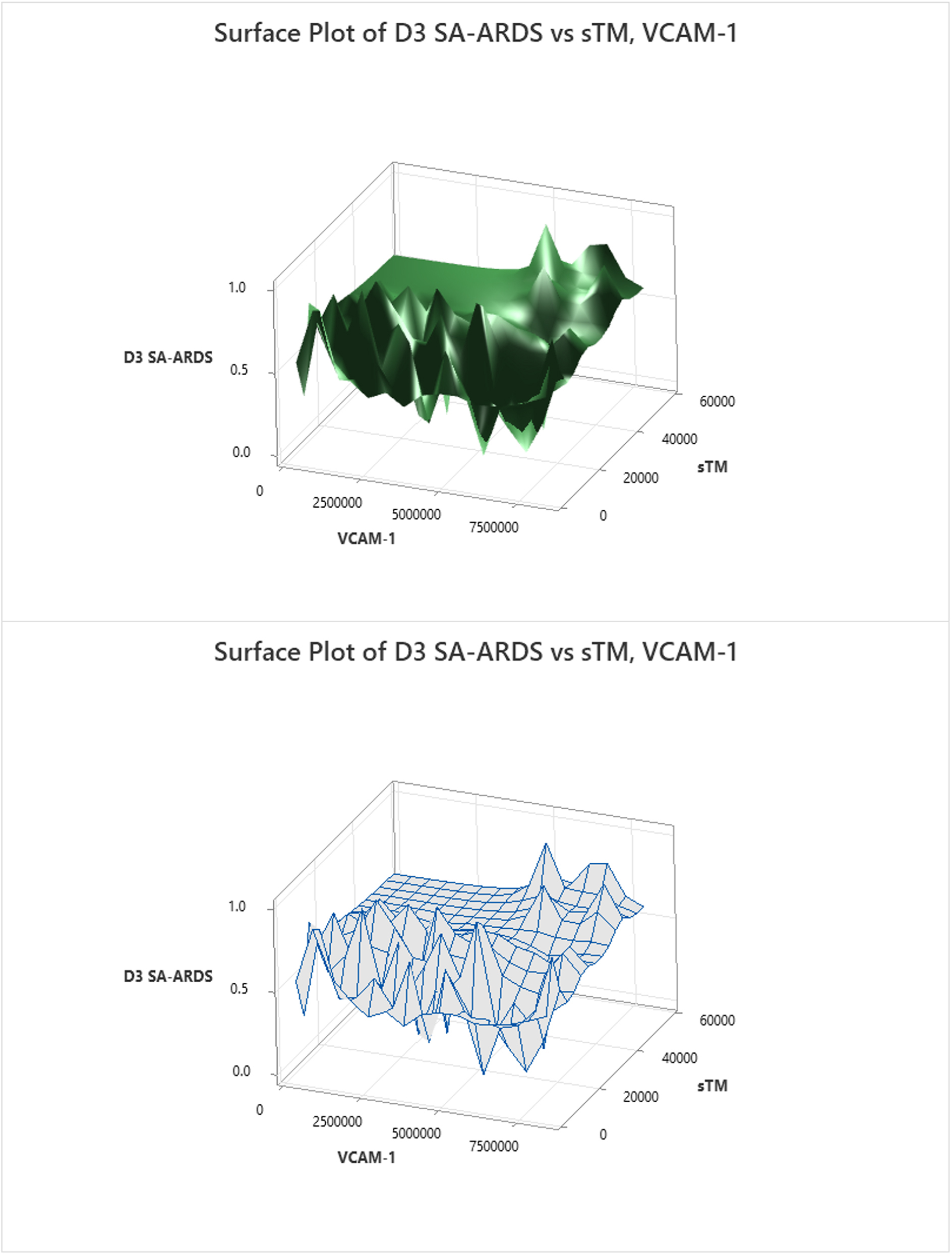

Supplement: fig6 [file NIHMS2066685-supplement-fig6.jpg]
